# In-Vivo Gene Therapy with Foamy Virus Vectors

**DOI:** 10.3390/v11121091

**Published:** 2019-11-23

**Authors:** Yogendra Singh Rajawat, Olivier Humbert, Hans-Peter Kiem

**Affiliations:** 1 Stem Cell and Gene Therapy Program, Fred Hutchinson Cancer Research Center, Seattle, WA 98109, USA; ohumbert@fredhutch.org (O.H.); yrajawat@fredhutch.org (Y.S.R.); 2 Departments of Medicine, University of Washington School of Medicine, Seattle, WA 98195, USA; 3 Departments of Pathology, University of Washington School of Medicine, Seattle, WA 98195, USA

**Keywords:** gene therapy, in-vivo gene therapy, hematopoietic stem and progenitor cells, foamy virus vector, pre-clinical canine model, SCID-X1

## Abstract

Foamy viruses (FVs) are nonpathogenic retroviruses that infect various animals including bovines, felines, nonhuman primates (NHPs), and can be transmitted to humans through zoonotic infection. Due to their non-pathogenic nature, broad tissue tropism and relatively safe integration profile, FVs have been engineered as novel vectors (foamy virus vector, FVV) for stable gene transfer into different cells and tissues. FVVs have emerged as an alternative platform to contemporary viral vectors (e.g., adeno associated and lentiviral vectors) for experimental and therapeutic gene therapy of a variety of monogenetic diseases. Some of the important features of FVVs include the ability to efficiently transduce hematopoietic stem and progenitor cells (HSPCs) from humans, NHPs, canines and rodents. We have successfully used FVV for proof of concept studies to demonstrate safety and efficacy following in-vivo delivery in large animal models. In this review, we will comprehensively discuss FVV based in-vivo gene therapy approaches established in the X-linked severe combined immunodeficiency (SCID-X1) canine model.

## 1. Introduction

Therapies based on gene transfer to hematopoietic stem and progenitor cells (HSPCs) have achieved tremendous curative outcomes over the past decade and due to revolutionary success in some of these gene therapy clinical trials, these outcomes will redefine the clinical management of patients [1,2,3]. Pioneering gene therapy trials have shown that the genetic engineering of HSPCs can be a potentially superior alternative to allogeneic transplantation in the treatment of hematological monogenetic disorders including primary immunodeficiencies [4,5,6]. Transfer of therapeutic genes into long-term repopulating HSPCs can potentially cure blood disorders such as hemoglobinopathies and primary immunodeficiencies. Specifically, with regards to X-linked severe combined immunodeficiency (SCID-X1), recent data showed that this approach could be curative in animal models [7,8] together with very promising clinical results using gene therapy in SCID-X1 patients [1,9]. Despite these advances, gene therapy continues to face a number of challenges which, if not resolved, could be detrimental to the clinical translation of these approaches [4]. These challenges range from the translation of research findings to clinical practice, covering issues with regards to the need for a conditioning regimen, vector-related genotoxicity, specific vector design, and the requirement of sophisticated manufacturing facilities, all of which present various obstacles towards efficacious and practically feasible gene therapy. Manipulation of HSPCs ex-vivo in clinical trials have several drawbacks including a cumbersome and expensive process of extracting and purifying HSPCs from the patient and returning the genetically modified cells to the patient, which also causes a delay in treatment. Further, relative refractoriness of human hematopoietic stem cells to clinically available vector systems remains a significant obstacle for most applications. To circumvent the current challenges and achieve practical HSPCs gene therapy, much work has focused on the development of newer delivery vehicles (viral vector based, or non-viral vector based using nanoparticles), alternative conditioning regimens (non-genotoxic), or simpler methods to target HSPCs in-situ by in vivo administration of the therapeutic transgene which bypasses the need for ex-vivo manipulation of these cells.

Priority should be given to the development of new viral vectors that overcome the known obstacles to stem cell transduction, such as the ability to transduce nondividing cells and utilization of virus that target receptors specific to primitive repopulating cells. With keeping in mind application and clinical translatability, various vectors such as γ-retroviral vector (γ-RVV, [9]), lentiviral (LVV, [1,10,11,12]), adenoviral [13,14,15,16] and adeno-associated viral [17,18] vectors have been designed to target refined cell populations with varying clinical and preclinical success. Although, significant progress has been made in the design of viral vectors, there are several limitations that still need considerable attention, particularly in the large-scale production of viral vectors under good manufacturing practice (GMP), which constitutes a major bottleneck. In recent times, AAV vectors have achieved clinical success; however, pre-existing immunity, relatively low transduction efficacy to non-dividing cells and relatively low transgene/cDNA (~4.4 kb) delivery capacity restricted their use [19,20]. Adenoviral vectors offer several advantages, including large cloning capacity and transduction of a number of tissues based on serotype, but their use has been restricted mainly due to their potent induction of acute immune response that could be fatal [21,22]. γ-RVV and LVV have shown very encouraging clinical outcomes specifically in monogenetic disorders of hematopoietic origin. However, the most commonly used envelope (VSV-G) to pseudotype clinical γ-RVV and LVV vectors targets HSPCs inefficiently and these vectors have significant batch to batch variability in large scale manufacturing and in their subsequent transduction of target cells [23,24]. Further, VSV-G pseudotyped lentiviral vectors are immunogenic, which leads to the elimination of corrected HSPCs in-vivo due to potent humoral and cellular immune responses, resulting in lower engraftment in patients [25,26]. To circumvent limitations of the currently available viral vector platform, there is an unmet need to either improve the current viral vector platform or to identify novel viral vectors applicable to HSPCs gene therapy. 

More than two decades ago, a new vector system based on foamy viruses from the spumavirus family was described that can be used for gene transfer into murine hematopoietic stem cells (HSCs) [27,28]. Foamy viruses are non-pathogenic retroviruses with a wide tissue tropism that are commonly found in mammalian species. David Russell and his group developed replication-defective foamy virus vectors (FVVs) and demonstrated that these vector particles efficiently transfer a marker gene into repopulating mouse HSCs and into human CD34+ cells ex-vivo [29,30]. Since then, our laboratory has optimized parameters for efficient transduction of human and canine CD34+ HSPCs by FVVs [31,32,33]. In the last decade, our group has demonstrated efficacy of FVV in correction of monogenetic diseases of hematopoietic origin in large animal models (non-human primates and canine) and has established the safety of FVV in-vivo [7,8,34,35]. In these pre-clinical studies, FVV-mediated transgene delivery maintained high and persistent levels of marker-gene expression possibly due to improved transduction of quiescent cells and to the novel envelope/receptor system used for stem cell entry [7,8]. In the following sections, we will discuss advantages of FVV for therapeutic gene therapy applications and will specifically focus on our studies using in-vivo FVV delivery for the treatment of canine model of SCID-X1 since general characteristics and different aspects of FV biology have already been discussed elsewhere in this special issue.

## 2. Limitations of Ex-Vivo Gene Therapy

Hematopoietic stem and progenitor cells ex-vivo gene therapy utilizing viral vectors have been used in multiple clinical trials to circumvent the complications associated with allogeneic bone marrow transplantation. Despite the undeniable therapeutic benefits offered by HSPCs gene therapy treatment for various monogenetic disorders such as hemoglobinopathies [36,37], primary immunodeficiencies [1,9,38,39] and inborn metabolic disorders [40,41], this approach poses several limitations which include requirement of cytotoxic conditioning regimen to promote engraftment of donor cells, lifelong administration of immunoglobulins in cases of immunodeficiencies, safety concerns due to vector genotoxicity (insertional mutagenesis), invasive procedure to procure HSPCs, and requirement of costly GMP facility for production of the cell product. Further, ex-vivo transduction protocols used in SCID-X1 clinical trials using SIN lentiviral vectors [1,42] required manipulation of HSCs by culture in multiple cytokines for approximately 3–4 days that may compromise HSCs pluripotency due to entry into pathways of differentiation and limit their engraftment potential and long-term repopulating capacity. Similar findings were reported in SCID-X1 dogs transplanted with bone marrow CD34+ cells from normal healthy donors [43]. Therefore, innovation is needed for long-term efficacy of ex-vivo gene therapies [44]. Alternatively, to circumvent these problems, an attractive approach is to transduce HSCs in-vivo within their natural environment by direct intravenous injection (in-vivo gene therapy). For comprehensive information of ex-vivo studies using FVV in HSPCs, we refer the reader to the excellent review by Vassilopoulos et al., in the same edition. In the current review, we will focus on FVV as an in-vivo gene therapy platform established in the canine model of SCID-X1. We will also discuss progress made in vector design and regimen used to mobilize HSCs out of the bone marrow.

## 3. Viral and Non-Viral Vectors Platforms for In-Vivo Gene Therapy

In lieu of various shortcomings of ex-vivo gene therapy, an alternative strategy that overcomes current limitations and still utilizes the benefits of gene therapy would provide a great advancement toward clinical translation. We propose that a possible answer lies in the use of in-vivo gene therapy. In-vivo gene transfer strategies administer the therapeutic vector either directly to the target organ or via the vascular system into blood vessels feeding that organ. In-vivo gene transfer offers several advantages over ex-vivo strategies including ease of administration, no need for HSPCs collection, manipulation and culture outside the body and thus no requirement for costly cell processing GMP facilities, and increased safety due to absence of myeloablative conditioning and transplantation procedures. Finally, this novel platform is portable and easy to disseminate worldwide particularly in under-developed countries with a large patient population needing treatment.

Multiple approaches and various delivery vehicles have been utilized for in-vivo transfer of therapeutic genes. These platforms include the use of non-viral [45] and viral vectors (integrating and non-integrating) [46] based approaches. Non-viral gene delivery systems have gained considerable attention as a promising alternative to viral delivery to treat various diseases [47,48]. However, despite extensive research, little is known about the parameters that underline the safe and effective in-vivo use of the nanoparticle-based delivery. So far, nanoparticles have shown promise in targeting and delivering cargo to various tumors very effectively. This success has been attributed to enhanced permeability and retention (the EPR effect) that can permit passive accumulation into tumor interstitial tissue. However, sub-optimal delivery is achieved with most nanoparticles because of heterogeneity of vascular permeability, which limits nanoparticle penetration. With regards to in- vivo gene therapy using nanoparticles, we cannot rely on passive accumulation and need to target tissue specific delivery of therapeutic cargo. Moreover, slow drug release limits bioavailability, which also restricts the use of nanoparticles for delivery of therapeutic cDNA. Overall, the modest efficacy, limited stability of nanoparticle conjugated to delivery cargo and lack of specificity of non-viral delivery are the central issues that need to be addressed. Thus, although nanoparticle-based approaches remain an attractive potential choice for in-vivo gene therapy, many questions still need to be answered for their effective clinical translation.

In early attempts with in-vivo gene therapy with viral vectors, VSV-G pseudotyped lentiviral vectors were used for direct intravenous injections in female rat brains using a stereotactic approach and showed effective transduction in multiple cell types including terminally differentiated neurons [49]. These early studies showed that LVVs can successfully be administered intravenously, transgene expression could be sustained for several months without detectable pathology, and they provided proof of concept which eventually led to multiple follow-up studies with various vector systems. Intravenous administration of early adenoviral vector platforms in ornithine transcarbamylase deficiency (OTCD) clinical trials [50] resulted in fatal systemic inflammatory response syndrome in one patient [22]. Further, the use of first generation γ-RVVs (non-SIN γ-RVV) derived from Moloney murine leukemia virus (MMLV) with duplicated viral enhancer sequences within the long terminal repeats (LTRs) led to leukemogenesis in early SCID-X1 clinical trials [51,52,53]. These early setbacks paved the path for the design of novel and safe viral vectors. In the past two decades, not only have there been inventions of a variety of safer viral vector such as “gutless adenoviral vectors” [54,55,56] and self-inactivating (SIN) γ-RVVs [9,57] and LVVs [12,58], but these vectors have also become the backbone of ex-vivo gene therapy clinical trials [24,59,60,61]. 

With regards to use of integrating viral vectors (γ-RVVs and LVV’s) in-vivo, intravenous administration of the retroviral replicating vector, Toca 511, recently demonstrated efficacy in orthotopic immune-competent mouse glioma model [62]. Further, a phase 1/2 study of a non-primate lentiviral vector based upon the equine infectious anemia virus (EIAV) expressing three genes involved in dopamine metabolism demonstrated safety of local lentiviral gene delivery into the central nervous system with evidence of clinical benefit [63]. In-vivo gene delivery using a lentiviral vector has also been applied clinically to the eye [64] and demonstrated that EIAV vectors provide a safe platform with robust and sustained transgene expression for ocular gene therapy. Cantore et al. reported the efficacy and safety of liver-directed in-vivo gene therapy in large and small animal models using lentiviral vectors. These vectors targeting the expression of a canine factor IX transgene in hepatocytes were well tolerated and provided a stable long-term production of coagulation factor IX in dogs with hemophilia B [65]. Even though these studies represent evidence of tremendous improvement in γ-RVV’s and LVV’s design for use as in-vivo gene delivery vehicles, major hurdles such as safety and host immune response against the vector and envelope used for pseudotyping restricts their use in clinical applications. Therefore, various strategies have been proposed to improve existing platforms to be utilized for in-vivo gene delivery [66,67,68]. 

Existing viral vectors have shown varying degrees of therapeutic efficacy in ex-vivo gene therapy clinical trials; nonetheless, little progress has been made for in-vivo clinical use with the exception of AAV vectors. In recent times, AAV vector-based in-vivo gene therapy has seen tremendous success for monogenetic disorders, which is evident with the recent approval of alipogene tiparvovec (Glybera, EMA, Amsterdam, Netherlands; year 2012) for the treatment of a rare inherited disorder, lipoprotein lipase deficiency, voretigene neparvovec (Luxturna, USFDA, Silver spring, Maryland, USA; year 2017) for the treatment of Leber’s congenital amaurosis and for the treatment of pediatric spinal muscular atrophy (SMA) with bi-allelic mutations in the survival motor neuron 1 (SMN1), geneonasemnogene abeparvovec-xioi (Zolgensma, USFDA, year 2019). Although AAV based approaches have seen clinical success, these vectors have several drawbacks including limited scope with regard to target tissues and cell types that do not divide rapidly. AAV is largely maintained episomally, with very limited vector getting integrated in genome that will limit long-term efficacy. Although AAV vectors have little or no acute toxicity, there are reports of development of hepatocellular carcinoma in the mice model [69], ocular toxicity in mice [70], and the use of AAV vectors resulted into severe toxicity in non-human primates and pigs [71]. Altogether, current viral vector-based approaches for in-vivo gene therapy need to be further improved by leveraging recent discoveries in viral biology, progress in vector design and transduction, or exploring the use of novel viral vectors. In the following section, we discuss our promising data using in-vivo administration of FVV’s for the treatment of SCID-X1 in the dogs. 

## 4. In-Vivo Gene Therapy for Canine SCID-X1 with FVVs

FVs are unique retroviruses which belong to Spumaretrovirus and are nonpathogenic to their natural host [72]. FVs are prevalent in many mammals including nonhuman primates but they are not endemic in human populations [73]. Cell membrane associated heparan sulfate is a receptor for the prototype foamy virus in many species including humans [74]. As heparan sulfate is expressed in a variety of cell types, FVs are able to infect many tissues. Prototype FVVs were developed owing to several unique properties including lack of pathogenicity [75], broad tropism (can transduce many therapeutic targets), large transgene capacity, unique replication strategy which provides the ability to persist in quiescent cells, safer integration profile [31,76,77] and resistance to serum complement inhibition [27,78] which is a determining factor for in-vivo gene therapy. FVVs system have evolved from early replication competent vectors to third generation non replicating viral vectors which are efficient gene delivery vehicles that have shown great promise for gene therapy in various preclinical animal models including our SCID-X1 dogs [79]. 

The SCID-X1 dog model provides a fantastic opportunity to delineate various therapeutic strategies that are very much translatable to human SCID-X1 patients. Our collaborators, Felsburg and colleagues, have established a SCID-X1 dog model in basset hounds breed in which immunodeficiency is caused by a naturally occurring genetic mutation in the common gamma chain (γC) [80,81]. The mutation is a four base pair frameshift deletion in the signal peptide region that results in a pre-mature termination codon in exon1 [82]. Unlike genetically engineered γC deficient mice, canine SCID-X1 has a clinical and immunologic phenotype representative of human SCID-X1, thus making it an ideal pre-clinical model to improve gene therapy strategies for human SCID-X1. 

**Figure 1:**
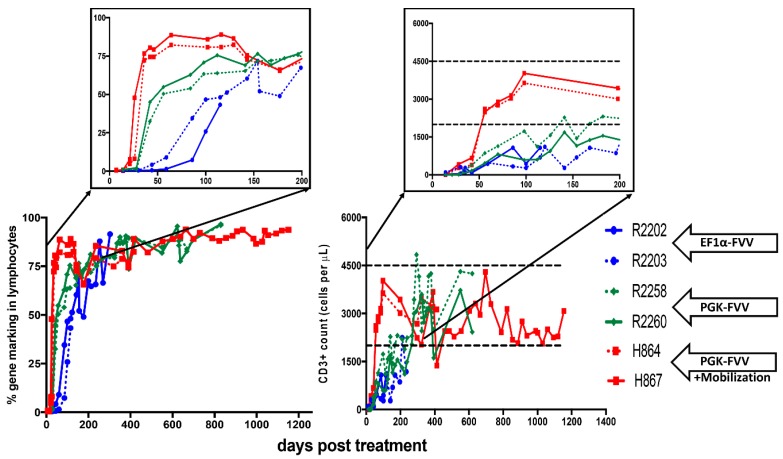
Immune reconstitution in foamy virus vector (FVV) treated X-linked severe combined immunodeficiency (SCID-X1) dogs: Bottom left graph shows % gene corrected lymphocytes in peripheral blood of various animals. Top left inset highlights the early kinetics of gene marking in treated animals (blue vs. green vs. red lines). The mobilized dog H867 had a stable level of gene marking almost 1260 days post treatment. Bottom right graph represents absolute number of CD3+ T lymphocytes in peripheral blood of treated dogs. Top right inset emphasizes the days required to attain normal numbers of absolute T lymphocytes (blue vs. green vs. red lines; dashed black lines shows counts in healthy dogs). H867 maintained normal levels of CD3+ T cell counts for over three years. Data in this figure was reproduced from previous studies [7,8] and contains extended data on H864 and H867. R2202 and R2203 were part of the cohort of five dogs from our first study [7] and R2258, R2260, H864 and H867 were part of our second study [8], additional details are included in the text. EF1α-FVV: elongation factor 1 α promoter (EF1α-GFP-2A-γC) carrying foamy virus vector (FVV); PGK-FVV: human phosphoglycerokinase (PGK-mCherry-γC) promoter carrying FVV.

In our very first study, we evaluated the efficacy of FVV gene therapy in treating SCID-X1 [7]. Five neonatal SCID-X1 dogs were treated by in-vivo administration of the FVV, containing Green Fluorescent Protein (GFP) and the coding sequence for the human common gamma chain (γC) linked by a 2A element, and expressed under control of the elongation factor 1 promoter (EF1α) (EF1α-EGFP-2A-γC). All five animals were intravenously injected with 4.0–8.4 × 10^8^ infectious unit of FVV (age at injection varied from one day old to 13 days old). The injection of FVV was well tolerated by all five pups with no adverse effect. γC+ lymphocytes were detected in peripheral blood within 14 days post-treatment and, by 84 days, γC+ cells comprised 30%–58% of the total lymphocytes. Four out of five dogs showed a parallel trend for GFP+ lymphocytes. While promising, these results were limited by the relatively slow rate of lymphocyte reconstitution in these animals. The dogs surviving long-term eventually recovered normal lymphocyte counts at 112 days post-treatment (R2202 and 2203, Figure 1 inset, blue lines, Figure 1 includes selected results of two dogs from first study [7]). GFP+ (i.e., gene corrected) lymphocytes eventually accounted for 73% to 91% of circulating lymphocytes and expression of γC was sufficient for the development of CD3+ T cells, comprising 7% to 43% of total lymphocytes in peripheral blood by six weeks after administration of FVV. As expected, the majority of CD3+ cells expressed GFP that originated from the gene therapy vector and stained positive for CD4 and CD8. Most of the CD3+ cells also stained positively for CD45RA, a marker for naïve T cells, indicating recent thymic emigration. 

We further assessed the T-cell receptor (TCR) diversity in each treated animal by spectratyping that analyzes genetic rearrangement of the 17 families of TCR Vβ segments. The longest surviving dog, R2202, initially expressed polyclonal TCR at early timepoints but eventually lost TCR diversity by 322 days post-treatment. These results demonstrated that delivery of the γC gene via FVV in-vivo in SCID-X1 dogs enabled thymocyte development and maturation as demonstrated by robust TCR rearrangement. Normal T cells counts as well as functionality of the γC-dependent signaling pathway were also restored, as demonstrated by tyrosine phosphorylation of the downstream STAT5 effector via activation of the γC pathway by IL-2 stimulation in peripheral blood mononuclear cells (PBMCs). Moreover, these γC+ lymphocytes were able to proliferate and re-enter into the cell cycle upon mitogen (phytohemagglutinin, PHA) stimulation as assessed by BrdU incorporation. Overall, FVV injection restored T-cell-mediated immunity with normal number and functionality of T cells generated. Specific antibody responses and immunoglobulin class switching was also evaluated in treated animals after immunization with the T cell-dependent neoantigen bacteriophage, ΦX174. This neoantigen is routinely being used in human patients with SCID-X1 to evaluate success of treatment with bone marrow transplantation or gene therapy. We found that treated animals showed a primary and secondary antibody response that is very similar to that seen in healthy human and canine subjects, indicating that our treatment restored both the B and T cell cytokine signaling required for class switching and memory responses to this neoantigen. 

To assess the safety and potential genotoxicity of FVV, retroviral integration site (RIS) analysis was performed longitudinally on peripheral blood of treated animals. Based on the identification of only 20 unique RISs across all samples, we concluded that all dogs displayed a polyclonal hematopoietic contribution in gene-modified cells over time. To determine if our in-vivo FVV treatment resulted in significant off-target activity (intended target cell population was bone marrow or blood derived HSCs or HSPCs), we assessed the biodistribution of the DNA provirus from various tissues by RIS. The majority of the identified integrants originated from the perfused blood into the tissues except for one event detected in the gut. We also found two integration events in the ovaries of R2202 but no integration was observed in the testis of R2203. Taken together, intravenous FVV gene therapy resulted in a very low frequency of off-target transduction events and are thus not likely to be passed on in the germline. Although clonal diversity and TCR repertoire were relatively low in these animals, these results provided proof of concept that FVV can safely be used in a pre-clinical model for in-vivo gene therapy. In conclusion, this first study demonstrated feasibility and safety of FVV in-vivo gene therapy in SCID-X1 dogs. Further, this study proved that in-vivo gene therapy using FVVs could achieve immune reconstitution in a clinically relevant large animal model of SCID-X1. 

## 5. Optimization of FVV In-Vivo Gene Delivery 

Our preliminary study using FVV, EF1α-EGFP-2A-γC was equally efficacious (in terms of T lymphocyte reconstitution) to earlier studies using in-vivo γ-retroviral vectors (γ-RVV) to treat canine SCID-X1 [83] or to ex-vivo γ-RVV clinical trial results reported in human patients [9]. However, this study demonstrated limited gene marking in the B and myeloid cell lineages as was reported in γ-RVV studies. In particular, treated animals still developed opportunistic infections (Table 1) and produced low immunoglobulin (Ig)G levels, and marking levels in granulocytes and monocytes were very low (0.6%), indicating that more efficient transduction of multipotent HSCs is required to achieve long-term phenotypic correction. 

The suboptimal immune reconstitution observed in the preliminary dog study prompted us to evaluate several strategies to further optimize our FVV in-vivo gene delivery protocol. The kinetics of immune reconstitution may be enhanced by modifying FVVs design, for example, by using a stronger promoter in place of the short form of the human EF1α promoter to drive expression of γC. In addition, targeting HSCs more efficiently may increase gene marking in other cell lineages that do not have a selective advantage like T lymphocytes. This could, in principle, be achieved by using mobilizing agents to increase the number of circulating HSCs in peripheral blood at the time of vector administration. 

In our next study using FVV for in-vivo gene therapy [8], we hypothesized that utilizing an alternative promoter to EF1α promoter could result in more robust γC expression in cells of hematologic origin. For this purpose, we redesigned our FVV with a human phosphoglycerokinase (hPGK) promoter to drive expression of the codon optimized human γC cDNA. Performance of each vector was compared side by side in a competitive repopulation assay by intravenous injection of equal doses of the FVVs, EF1α-EGFP -γC and PGK-mCherry -γC, in two newborn SCID-X1 animals. Competitive injection of EF1α-EGFP-γC and PGK-mCherry-γC in the same SCID-X1 dog bestowed an ideal opportunity to study the efficacy of each promoter under similar physiological conditions in the same animal. The absolute number of circulating lymphocytes steadily increased in both treated dogs during the first six months post-treatment, plateaued around 6–8 months, and remained within the normal range during the course of 2.5 years post-treatment (dogs R2258 and R2260, Figure 1, Green Lines). Strikingly, the majority of gene marking (70% to 90%) in peripheral blood came from the PGK-mCherry-γC vector in both animals, whereas marking from the EF1α-EGFP-γC vector comprised only a small fraction (5% to 10%). Interestingly, the early kinetics of gene marking in peripheral blood lymphocytes in these two animals was substantially improved as compared with animals treated with the EF1α-EGFP-γC vector from our previous study (R2202 and R2203, Figure 1). The fraction of gene-corrected peripheral lymphocytes reached 40% in both R2258 and R2260 at six weeks post-injection, as compared with 5% for the EF1α-EGFP-γC alone treated animal, demonstrating superior therapeutic performance of the PGK-mCherry-γC vector as compared to EF1α-EGFP-γC. Nevertheless, this new vector did not result in improved targeting of the most primitive HSCs as showcased by limited gene marking in non-T cell lineages (B and myeloid cells). 

In an attempt to target HSCs more effectively, we chose to treat a new animal cohort with a mobilization agent to increase the frequency of HSCs in peripheral blood at the time of FVV administration. Stem cell mobilization is defined as a process in which certain drugs are used to cause the trafficking of stem cells from the bone marrow into the blood and is commonly used to collect and store stem cells that may be used later as for bone marrow replacement therapy during a stem cell transplant. Granulocyte colony-stimulating factor (G-CSF) mobilized HSPCs from peripheral blood is the most widely used source of HSPCs for clinical transplantation [84]. As an alternative, plerixafor (AMD3100) was shown to not only efficiently mobilize the HSPCs in various species including humans [85,86], but also augment the mobilization and yield of CD34+ HSPCs when used in combination with G-CSF for clinical transplantation [87,88]. Furthermore, plerixafor was found to be very effective at mobilizing CD34+ HSPCs in dogs (3–10 fold increase in circulating CD34+ HSPCs count) [89]. Therefore, we hypothesized that mobilization prior to injection of FVV may enhance HSPCs transduction efficiency in-vivo. In the next cohort of animals, we treated two SCID-X1 dogs with both plerixafor (4 mg/kg, subcutaneously, single dose) and G-CSF (5 μg/kg, subcutaneously, twice a day for five days), which resulted in a 6.4–7.2-fold increase in circulating CD34+ cells [8]. Plerixafor treatment significantly increased the kinetics of lymphocyte expansion and gene marking as compared to non-mobilized PGK-mCherry-γC-treated animals. The fraction of gene-corrected lymphocytes in peripheral blood of mobilized animals reached 80% at six weeks post-treatment, whereas it took >20 weeks in non-mobilized animals to reach similar levels (green lines vs. red lines comparison in Figure 1). Accordingly, the time required to reach normal lymphocyte counts was markedly reduced in the mobilized animals (red lines, Figure 1). The two non-mobilized FV vector-treated animals (R2258 and R2260) initially showed normal frequency (90%) of CD3+CD45RA+ T cells in peripheral blood, but their frequency subsequently declined to 50% at one-year post-treatment. In comparison, levels of CD3+CD45RA+ cells have remained stable for both mobilized dogs, up to 18 months in H864 and for over 36 months post-treatment in H867, which continues to be monitored. 

Thymic output was assessed by analysis of T cell receptor excision circles (TRECs) originating from TCR genes rearrangement that occurs during T-lymphocyte maturation. In the non-mobilized animals, TRECs were initially 10-fold lower in treated dogs (1000–1200 TREC/million peripheral blood mononuclear cells, PBMCs), as compared with a normal littermate control (12,000–14,000 TREC/million PBMCs), and then gradually declined over time. In contrast, TRECs in the mobilized animals reached normal levels as early as three months post-treatment and remained similar to the littermate control for up to three years post-treatment. In summary, we found that mobilization before FVV injection of SCID-X1 canines improved kinetic of T-lymphocyte reconstitution and increased thymic output to levels comparable to those in a healthy control. 

The majority of expanded CD3+ lymphocytes were mature and expressed the coreceptor CD4 or CD8, with a small fraction of cells being CD4/CD8 double positive or double negative. Both mobilized animals H864 and H867 showed normal CD4:CD8 cell ratios, averaging two. The majority of circulating T lymphocytes in non-mobilized (R2258/R2260) and mobilized (H864/H867) also stained positive for TCR α/β starting at two months post-treatment, consistent with observations from healthy canines and humans. When assessing TCR diversity in each treated animal by TCR β spectratyping, we found that the two animals mobilized with G-CSF/AMD3100 showed robust spectratype profiles comparable to that of an aged-matched normal littermate, characterized by Gaussian distribution of fragments sized across 17 families of TCR vector β segments, and stable for up to three years post-treatment. 

Similar to what we described in our previous study, we also verified functionality of the γC-dependent signaling pathways in all FVV-treated animals as well as effective stimulation in response to T-cell mitogen phytohemagglutinin. Primary and secondary antibody responses, and immunoglobulin class switching after immunization with the T cell–dependent neoantigen bacteriophage ΦX174 was also documented in these animals. In addition, polyclonal IgM, IgG, and IgA concentrations were measured from serum of mobilized dogs at multiple timepoints post-treatment and showed IgG (1850–3152 mg/dL) and IgM levels (250–382 mg/dL) in the treated SCID animals that were comparable to a healthy littermate control (IgG: 670–1650 mg/dL: IgM: 100–400 mg/dL), indicating partial restoration of B-lymphocyte function. Although, antibody levels were within normal range for the mobilized FVV treated dogs (H864 and H867), the gene marking levels in B lymphocytes were low throughout the study. In fact, despite substantially improving T-lymphocyte reconstitution, HSPC mobilization did not increase gene marking in myeloid cells (0–1.5%) or B lymphocytes (0–4%) in mobilized dogs, which is consistent with the levels of gene marking in myeloid and B lymphocytes in non-mobilized dogs. Thus, FVV in-vivo gene therapy can result in low levels of correction of HSCs or myeloid progenitors in addition to circulating common lymphoid or T cell progenitors that experience a selective growth advantage after gene correction.

To assess the safety and potential genotoxicity of FVVs, tissues from non-mobilized animals (R2258 and R2260) were collected and analyzed by RIS for biodistribution assessment of the foamy provirus. The vast majority of RISs (90%) detected in tissues were also found in peripheral blood at the same time point, suggesting that they originated from contaminating blood cells present in perfused tissues. Interestingly, ovaries and testes showed the smallest number of integration events (37 and 56, respectively, as compared with 766 and 469 in blood), and none of the RISs found exclusively in the gonadal tissues appeared at notable frequencies except for one integration site in the ovaries (chromosome 38; 34,522; 4.28%). No unique RIS at a noteworthy frequency was detected in semen from mobilized male H867. Taken together, these results suggested that off-target transduction events by in-vivo FVV treatment are rare and thus unlikely to be passed on in the germline, a finding also supported by the study of progeny issued from FVV-treated male R2260. RIS analysis from peripheral white blood cell DNA showed a marked increase in integration events in mobilized dogs H864 and H867 as compared with non-mobilized dogs R2258 and R2260, despite the use of an equal dose of FVV PGK-γC, consistent with the greater therapeutic activity of this vector. No clonal dominance was observed in any animal, but some persisting clones contributing to 0.1% of total gene marking were found in the non-mobilized animals, albeit with no indication of expansion. Taken together, our studies indicated that the use of G-CSF/AMD3100 mobilization before intravenous FVV delivery increases both the kinetics of lymphocyte recovery and diversity of immune reconstitution in SCID-X1 canines. 

Altogether, we have so far treated nine dogs (five with EF1α-EGFP-2A-γC; two with both EF1⍺-EGFP-2A-γC and PGK-mCherry-2A-γC and two dogs with PGK-mCherry-2A-γC and G-CSF/AMD3100 mobilization) [5,6] and all treated dogs demonstrated efficacy and safety of FVV in-vivo gene therapy in canine model. Out of nine treated SCID-X1 dogs, five lived more than a year and three lived for over 2.5 years. Most importantly, the kinetics of lymphocyte reconstitution in our study using PGK-mCherry-2A-γC and G-CSF/AMD3100 mobilization is comparable that of SCID-X1 patients treated using ex-vivo gene therapy [9]. The long-term treated dogs demonstrated that correction of cellular and humoral immune compartment is sustained for over three years. Even though we have seen therapeutic correction of SCID phenotype in the dogs, particularly in T cell immune reconstitution, there is still room for improvement in myeloid and B cell gene marking. This could be achieved by treatment with more effective HSC mobilization regimen, by directly targeting primitive HSCs in their niche through intra-osseous delivery [90] of the viral vector, or with the use of a selection strategy for gene-modified HSCs [34,91]. Our findings from FVV-treated dogs are directly translatable to human SCID-X1 patients and validate FVVs as an effective vehicle for in-vivo delivery of the therapeutic transgene to correct SCID-X1 and potentially for other hematologic disorder. 

## 6. Summary and Future Perspective

Ex-vivo HSPCs gene therapy clinical trials using non-SIN γ-RVV, SIN γ-RVV and LVV for SCID-X1 patients have demonstrated tremendous clinical success and will change the current practice of patient care. However, these therapies still require, in most cases, high doses of conditioning with chemotherapy, and thus patients are myelosuppressed for prolonged periods requiring hospitalization. In addition, conditioning can lead to genotoxicity and secondary malignancies. All ex-vivo approaches require invasive procedures to procure HSPCs and appropriate facilities, with very high cost. Due to these limitations, it will be challenging to apply current ex-vivo gene therapy strategies using integrating viral vectors on a broad scale. Therefore, there is an unfulfilled and ongoing quest for a safer and affordable treatment option for SCID-X1 patients. In-vivo gene therapy offers several advantages and could be a potential alternative to mitigate some of the challenges seen with ex-vivo HSPCs gene therapy. In terms of in-vivo gene therapy, γ-RVV and LVV have been used pre-clinically for disease models (as discussed briefly in this review) other than SCID-X1, with varying degree of therapeutic efficacy. However, challenges such as safety and immunogenicity remain a major hurdle for clinical translation of these vectors involving in-vivo delivery.

In-vivo gene therapy with FVVs has provided encouraging long term safety and efficacy results in the pre-clinical SCID-X1 dog model. These findings demonstrate comparable efficacy in terms of immune reconstitution and T cell functionality to human SCID-X1 clinical trials with the use of γ-RVV and LVV in ex-vivo gene therapy. Interestingly, in our SCID-X1 model, we have seen production of immunoglobulins that show normal B cell function with no prior conditioning, whereas, in human clinical trials normal function of B cells and production of immunoglobulin’s (IgG and IgA) was attributed to use of conditioning regimen. Therefore, FVV’s could provide an alternative platform for in-vivo gene therapy to mitigate some of the challenges possessed by γ-RVV and LVV. Our current pre-clinical FVV in-vivo gene therapy offers a path forward as an effective, safe, and accessible platform that may provide prompt treatment of newborn SCID-X1 patients without the complications associated with conditioning or manipulation of HSPCs. Moreover, this treatment scheme could be applied in other hematological disorders with monogenetic mutations, particularly in those disorders where manipulation of HSPCs ex-vivo is near impossible and where transfer of the therapeutic gene in few stem cells could be curative such as Fanconi anemia. 

The excellent therapeutic benefits reported in patients and regulatory approval of viral vector-based gene therapy products such as Glybera, Luxturna, and Zolzensma are providing enough impetus to continue the exploration of novel in-vivo gene transfer approaches. However, the choices of viral vector for in-vivo gene therapy will depend on specific disease, target tissue in hand, size of transgene delivered, and packaging capacity of vector. Specific challenges that need to overcome for in-vivo gene transfer strategies include the induction of immunity by the viral vector, access of the gene therapy vector to the targeted cells/organ, efficient targeting of the vector to the cell and translocation of the genetic material to the nucleus, and any toxicity induced by expression of virus and/or transgene. Further, ideal in-vivo gene therapy that should be affordable as Glybera (now withdrawn from market), Luxturna, and Zolzensma are very expensive and could be a concern for widespread usage and commercial interest. Among the integrating viral vectors, FVV provides a suitable platform due to several advantages offered as compared to γ-RVV and LVV as discussed in this study. With our recent pre-clinical data in the canine model of SCID-X1, FVV have so far successfully shown clear long-term safety and therapeutic efficacy. This portable in-vivo gene delivery platform circumvents some of the challenges imposed by some clinically used viral vectors. Importantly, with the advent of novel gene-editing approaches, FVV, in its engineered integration-deficient form, provides an attractive option for the in-vivo delivery of editing reagents (Cas9, gRNA and donor template) due to its large packaging capacity. Altogether, FVV could be an answer to some of the challenges faced today in clinical translation via the in-vivo delivery of gene therapeutics.

## Figures and Tables

**Table 1:** Description of SCID-X1 dogs treated by intravenous injection of FVV in various in-vivo gene therapy studies [7,8]. Two out of five dogs from the EF1α-EGFP-2A-γC study [7] were selected for inclusion in the table.

ID	Age at Injection (Days Old.)	Foamy Viral Vector	Dose of Vector (Infectious Units)	Mobilization	Survival of Dogs (Days Post Treatment)	Health Status or Infectious Complications
H867	16	PGK.mCherry.2A.γC	4.0 × 10^8^	G-CSF/AMD3100	1260	Healthy and Alive
H864	16	PGK.mCherry.2A.γC	4.0 × 10^8^	G-CSF/AMD3100	~486	*Bordetella bronchiseptica*
R2258	18	EF1⍺.EGFP.2A.γC	4.0 × 10^8^	NO	~820	Papillomavirus
		PGK.mCherry.2A.γC	4.0 × 10^8^			
R2260	18	EF1⍺.mCherry.2A.γC	4.0 × 10^8^	NO	~820	Papillomavirus
		PGK.EGFP.2A.γC	4.0 × 10^8^			
R2202	1	EF1α-EGFP-2A-γC	4.2 × 10^8^	NO	~334	Coccidiosis; Canine Distemper virus
R2203	1	EF1α-EGFP-2A-γC	4.2 × 10^8^	NO	~120	Canine Parainfluenza virus
